# The 2001-03 Famine and the Dynamics of HIV in Malawi: A Natural Experiment

**DOI:** 10.1371/journal.pone.0135108

**Published:** 2015-09-02

**Authors:** Michael Loevinsohn

**Affiliations:** Institute of Development Studies at the University of Sussex, Brighton, United Kingdom; Vanderbilt University, UNITED STATES

## Abstract

**Background:**

Food security has deteriorated for many people in developing regions facing high and volatile food prices. Without effective and equitable responses, the situation is likely to worsen due to diminishing access to land and water, competition from non-food uses of agricultural products, and the effects of climate change and variability. Understanding how this will affect the burden and distribution of major diseases such as HIV is critical. This study makes use of the near-experimental conditions created by the Malawi famine to shed new light on this issue.

**Methods:**

Multilevel, random intercept models were used to relate the change in HIV prevalence at antenatal surveillance sites over the course of the famine to the proportion of rural households requiring food aid in the surrounding district at the famine’s peak. Similar models were used to relate this indicator of rural hunger to changes in the composition of the antenatal population. The extent and direction of migration were estimated from a household survey conducted 1–2 years after the famine.

**Findings:**

At rural sites, the change in HIV prevalence was positively and non-linearly related to the extent of rural hunger (P = 0.016), consistent with contemporary accounts of increased transactional sex and with hunger compromising immune function. At non-rural sites, prevalence declined as rural hunger increased (P = 0.006), concentrated in women who self-identified as farmers (P = 0.010). This finding is consistent with contemporary accounts of migration in search of food and work from villages where HIV risk was lower to towns and cities where it was higher. Corroborating this interpretation, the proportion of farmers in the antenatal population was found to rise at non-rural sites as rural hunger increased in the surrounding district (P = 0.015) whereas the proportion fell with increasing rural hunger at rural sites (P<0.001). The models suggest migrants were predominantly farming women under 25 years (P = 0.010). The household survey confirmed that there was a surge of rural-to-urban migration during the famine, particularly by women under 25 years. Migration to less affected rural areas also increased.

**Conclusion:**

The Malawi famine appears to have had a substantial effect on HIV’s dynamics and demography. Poverty and inequality, commonly considered structural determinants of HIV epidemics, can change rapidly, apparently transmitting their effects with little lag. Epidemic patterns risk being misread if such social and economic change is ignored. Many studies examining HIV prevalence declines have implicated sexual behaviour change but do not appear to have adequately considered the contribution of rural-urban migration. The evidence from Malawi, which links actions that undermined people’s food security to changes in the prevalence and distribution of HIV infections, suggests new opportunities for prevention.

## Introduction

Large numbers of people in developing countries are regularly without secure access to food [1]. Surges in food price have put many others at risk of hunger and malnutrition. Without effective and equitable responses, these risks are likely to increase due to diminishing access to land, water and other key resources, competition from non-food uses of agricultural products, and the effects of climate change and variability [2]. Most vulnerable are consumers and small farmers in countries where farming is predominantly rainfed [3].

The impact of rising levels of hunger on human health and major diseases is of wide concern, however our ability to predict their impact is limited. With respect to HIV, much of what is known about the effect of hunger and other facets of poverty comes from cross-sectional or longitudinal studies of limited duration that shed little light on dynamic effects, whether trends, cycles, or major shocks [4–6]. The fact that hunger is both a cause and a consequence of HIV further limits the ability of these methods to disentangle the effect of change in either of them.

This paper brings new light to bear on this question. It assesses the 2001–03 famine in Malawi as a country-scale natural experiment on the effect of hunger on the dynamics of HIV. As described below, the famine “intervention” was “sharp, well-defined but unplanned” and its effects were unequally experienced among rural areas, between rural and non-rural areas and between men and women—characteristics of a natural experiment [7]. However, the Malawi famine was a consequence largely of actions and policies that, as in many famines, restricted people’s access to food [8]. There was, I emphasize, nothing natural in an entirely avoidable tragedy that took thousands of lives and disrupted many others. Analysing the famine as an experiment may provide new insights into HIV’s dynamics and ideas as to how it can be better prevented, discussed in the final section.

### The famine and HIV risk

The roots of the famine can be traced to under-investment in agriculture and erosion of dry season livelihood options in the preceding decades. These left rural Malawians vulnerable to the fate of the main maize harvest and increasingly dependent on casual agricultural labour (“*ganyu*”) and the market to supplement their own production [9–11]. Drought and poor but unexceptional harvests in 2001 and 2002 forced more people into the search for a diminished availability of work, further depressing wage rates. Ill-considered institutional responses contributed to a steep rise in prices: by February 2002, a kilo of maize cost 2–6 times what it had the previous year, in most markets considerably more than a labourer’s daily wage [12–15]. Although the contexts differed, similar food crises affected five other southern African countries: Zambia, Zimbabwe, Mozambique, Lesotho and Swaziland [16]

Deaths from hunger and cholera were reported in several parts of Malawi late in 2001 [12,17]. Nation-wide surveys in late 2002 found that 31% of rural households would need food assistance between December 2002 and March 2003, the traditional lean period before the next harvest, unevenly spread but substantial in all regions. Hunger was less severe among wealthier households, those headed by men and those with non-agricultural income [18,19]. Some of these profited from the situation, offering *ganyu* for lower wages and buying assets that farmers sold in distress. Households in the towns and cities, where maize was more reliably available and its price less volatile, were also less affected by hunger, reinforcing existing economic and social disparities between urban and rural areas [12,20].

As the famine intensified, rural Malawians increasingly resorted to measures that many, particularly the most vulnerable, had come to rely on in the annual lean season, for example, regularly going entire days without eating and removing children from school. Some of these actions increased their exposure to HIV. Among these was transactional sex—women selling or exchanging sex for food or gifts on an occasional basis [21,22]. Fifteen percent of young women and 6% of older women surveyed in 10 districts acknowledged exchanging sex for food during the crisis [23], likely underestimates given the difficulty assessing this sensitive matter through formal interviews. In the Central region, sexual services were frequently demanded of women, single and married, seeking *ganyu*, a practice that continued after the famine [24,25]. Reports also emerged during the famine of girls and young women being forced into marriage with older men [23,26]. They would have been at heightened risk of HIV and STIs because their partners were more likely to be infected than men their age and more likely to subject them to physical or sexual violence [27,28].

Greater involvement in transactional sex and early marriage would be expected to increase HIV incidence and prevalence in rural women in proportion to the extent and duration of hunger locally. Early marriage’s effect would be concentrated in younger women while that of transactional sex, which apparently involved both married and single women, would be more even. The unequal experience of hunger by rural men and women may also have contributed to more asymmetrical relationships (e.g. a man able to provide *ganyu* having sex with several workers) and concurrent relationships (e.g. the man maintaining relationships with his wife and one or more workers). Both have been shown to hasten HIV transmission and the latter to increase peak prevalence [29–31]. Malnutrition itself may have increased women’s risk of infection once exposed to the virus by suppressing immune function. The evidence for this is clearest with respect to sexually transmitted and parasitic infections that facilitate HIV transmission [32].

Many adults also moved in search of food or work to towns, cities, neighbouring countries and less affected rural areas [16,22]. Among 1,200 rural households surveyed in 2002, 39% had adult members migrating [33]. People moving in distress often face poor working and living conditions at their destination. Separated from partners and family, migrants in general are more likely to engage in non-marital sex and to be HIV-infected than are non-migrants [34–36]. The impact of hunger-induced migration on HIV prevalence in Malawi depends on how long people remained away from home. There has till now been no information on this but evidence from other famines suggests that not all migration is temporary [37]. To the extent that was true in Malawi, one would expect to see changes in the distribution of HIV and population in both source and destination areas.

In summary, the famine is hypothesized to have increased three of the fundamental determinants of HIV prevalence: incidence, emigration and immigration in rural villages; and immigration and possibly subsequent incidence in towns and cities.

The eminent epidemiologist Mervin Susser suggested that the term natural experiment be reserved for “observations of the effects of … events that are major, sharp, and out of the ordinary.[38]” It is entirely appropriate to assess the famine’s effects on HIV’s dynamics in this way. It was the most severe in the historical record, estimated to have caused the deaths of up to 85,000 people, nearly 1% of the rural population [14]. Many Malawians referred to it as *chinkukuzi*, the time “no one will survive” in Chichewa [39]. It is not just in retrospect that the questions at the heart of this paper have been asked. Health and development researchers working in Malawi at the time, myself included, were deeply concerned about the potential of sharply rising maize prices to accelerate the spread of HIV [21] and joined with colleagues in the region in a program of action research on HIV, AIDS and the southern African food crises [40].

## Methods

### Data

The prevalence of rural hunger in the district surrounding HIV surveillance sites was derived from a country-wide humanitarian survey in August 2002 (1128 households, stratified by wealth, in 81 villages). Whether a household would experience a significant cereal deficit in December 2002–March 2003, the lean season in the famine’s second year, was estimated using a model-based index that weighed 10 factors, including the number of members, their food requirements, cereal stocks, anticipated production and income. Household estimates were then aggregated to district level. [18]. For Mzuzu city, which is not part of any district, the average of the estimates for the two districts that surround it is used.

Estimates of the prevalence, timing and direction of migration were calculated from a country-wide survey in 2004 and early 2005 (n = 10,777 households) which asked when and from where members had moved to their present residence [41].

HIV prevalence estimates from before and after the worst of the famine were drawn from the antenatal surveillance rounds in 1999/2000 (median sample date 7/1/2000, 15%–85% completed 30/12/1999–24/1/2000), approximately 18 months before the steep rise in maize price, and 2003 (median sample date 11/3/2003, 15%-85% completed 16/2/2002–13/5/2003) overlapping the lean season in the famine’s second year and the post-harvest period when food was more readily available (S1 Text, *see web appendix*). (The 2001 round cannot be used because it overlaps the rise in maize price). Eight of the 19 sites are rural, located in village health centres and 8 are semi-urban, located in hospitals in district administrative centres (*bomas*). These sites were established in randomly selected districts, stratifying for region. One semi-urban site, Nkhotakota, is discarded because its results in 2003 were unreliable [42]. Three sites are urban, located in hospitals or health centres in the three largest cities, Mzuzu, Lilongwe and Blantyre.

**Fig 1:**
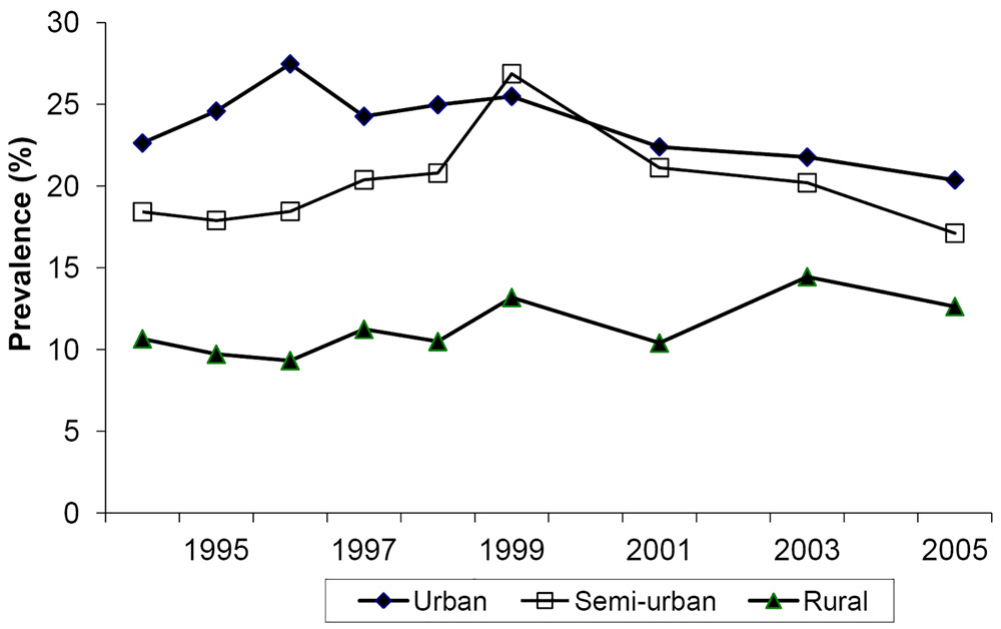
Prevalence of HIV in women attending antenatal clinics in Malawi 1994–2005. Unweighted means. The number of sites was constant over this period. Data courtesy Malawi National AIDS Commission.

Antenatal surveillance is the principal method for monitoring tends in HIV infection in countries where the epidemic is generalized [43] and provides a reasonable estimate of prevalence among women in the catchment area of surveillance sites [44]. In Malawi, procedures were consistent in these two rounds and followed international guidelines. On each round, a consecutive sample of women on their first antenatal visit were tested anonymously for HIV using a single enzyme-linked immunosorbent assay. Their age, education status, occupation and partner’s occupation were also recorded [42]. In the following analyses, women are considered as either farmer or non-farmer: a woman is classed as a farmer if she gave her or her partner’s occupation as “farmer”. Rural clinics attract predominantly farmers, urban clinics mostly non-farmers and semi-urban clinics intermediate proportions (Table 1). Fig 1 shows the trends in HIV prevalence since 1994. Prevalence has been similar at urban and semi-urban sites since 1999/2000 and are analysed together as “non-rural”.

**Table 1:** Characteristics of the women surveyed at antenatal surveillance sites and of the surrounding district.

		Rural (8 sites)		Non-rural (10 sites)			
Variable	Category			Semi-urban (7 sites)		Urban (3 sites)	
		1999 (n = 1144)	2003 (n = 1627)	1999 (n = 3392)	2003 (n = 3618)	1999 (n = 1901)	2003 (n = 2460)
**HIV serostatus**	**Positive**	12.1 (2.4)	14.5 (2.1)	27.6 (2.0)	21.6 (2.3)	25.6 (1.4)	21.8 (3.1)
	**Negative**	88.9 (2.4)	86.4 (2.1)	72.4 (2.0)	78.4 (2.3)	74.4 (1.4)	78.2 (3.1)
**Age**	**15–19 yrs**	24.9 (3.1)	22.0 (2.4)	24.0 (1.7)	20.7 (0.7)	20.0 (1.6)	19.7 (0.6)
	**20–24 yrs**	38.9 (3.1)	36.2 (1.0)	39.5 (1.0)	40.3 (1.6)	43.2 (2.3)	44.1 (1.2)
	**25–44 yrs**	36.2 (2.6)	41.8 (2.2)	36.5 (1.8)	39.1 (2.1)	36.8 (2.3)	36.2 (1.8)
**Education**	**None**	23.6 (4.7)	29.8 (6.7)	29.1 (7.6)	31.7 (10.7)	8.8 (2.7)	10.2 (3.2)
	**Primary**	70.6 (3.8)	64.6 (6.6)	58.6 (6.2)	54.8 (8.3)	62.4 (5.8)	63.9 (1.6)
	**Secondary +**	5.8 (1.1)	5.7 (0.9)	12.3 (1.6)	13.4 (2.6)	28.8 (6.3)	25.9 (3.5)
**Occupation**	**Farmer**	76.9 (5.1)	77.2 (4.6)	46.5 (3.5)	40.7 (6.3)	5.9 (4.3)	5.7 (2.9)
	**Non-farmer**	23.1 (5.1)	22.8 (4.6)	53.5 (3.5)	59.3 (6.3)	94.1 (4.3)	94.3 (2.9)
**Rural population in need of food assistance Dec 2002-March 2003**		32.6 (3.8)		26.0 (4.3)		20.8 (3.8)	

Data are percent (se).

### Analytical methods

Multilevel, random intercept models are used to analyse the contribution of contextual factors at the surveillance site level (rural or non-rural setting, the extent of rural hunger in the district) and compositional factors at the individual level (age, occupation and education) and their interactions, to the probability of a woman being HIV-infected and to the change in this probability over the course of the famine. The serostatus of the *i*th woman in the *j*th site in 2003, *y*_*ij*_, is a binary variate that takes the value 1 (positive) with probability *π03*_*ij*_. The log link relates this probability to a linear function of factors and covariates. Random variation at the individual and site levels is assumed to follow binomial and normal distributions, respectively. A site level model is first estimated:

$$\text{log}\pi 03_{ij}=\text{log}\pi 99_{j}+\beta_{0j}+\beta_{1}\cdot rural_{j}+\beta_{2}\cdot rural_{j}\cdot hunger_{j}+\beta_{3}\cdot nonrural_{j}\cdot hunger_{j}+\mu_{0j}$$ (1)

where log*π03*_*ij*_ is the log probability of a woman being positive in 2003 and log*π99*_*j*_ the mean log probability of being positive for all women at the *j*th site in 1999/2000. Subtracting the latter term from both sides makes the dependent variable the log relative risk of being HIV infected in 2003 vs. 1999/2000 (adapting an approach suggested by Ukoumunne and Thompson [45]). *β*_*0j*_ is the fixed part of the intercept and *μ*_*0j*_ its site-specific random effect. *rural*_*j*_ and *nonrural*_*j*_ are dummy variables taking the value 1 when the site is rural or non-rural, respectively, and 0 otherwise. *β*_*1*_ is thus the additional fixed part of the intercept at rural sites, while *β*_*2*_ and *β*_*3*_ are the linear coefficients of rural hunger in the district surrounding rural and non-rural sites, respectively. The *hunger*_*j*_ variable was arc sine-transformed and centred on its overall mean. Quadratic terms in *hunger*_*j*_ were also fitted to test for non-linear relationships.

Model 2 adds the individual-level factors and their interactions with rural hunger. The coefficient associated with a class of a factor, e.g. farmer in occupation, indicates the extent to which change from the 1999/2000 mean log probability of infection was greater or less for farmers than for non-farmers. The coefficient associated with the interaction term indicates the extent to which the difference between farmers and non-farmers varied with the level of rural hunger in the district. In both cases, the coefficients control for other individual and site-level variables.

Change through the famine in the composition of women sampled at the sites was estimated with models 3 and 4, of the form of models 1 and 2 but with log relative risk of a woman being a farmer in 2003 as dependent variable.

A model (5) with the individual-level factors and site terms, but without hunger, was estimated to assess determinants of prevalence in 2003.

These models were estimated with the GLLAMM program [46] in STATA 11 (StataCorp), employing maximum likelihood estimation with adaptive quadrature. However, log-binomial regression is known to yield biased estimates when the probability *π* is large (> 0.8) [47], as it is for the proportion of farmers at several sites. Models 1–4 were therefore also estimated using logistic regression so that the dependent variable becomes the log odds ratio of being infected or of being a farmer in 2003 vs. 1999/2000. This procedure avoids the estimation problem but yields a measure of the impact of exposure on risk, the odds ratio, that is less intuitive than the relative risk [48]. Results were confirmed by Monte Carlo Markov Chain (MCMC) simulation, which provides more precise parameter estimates with small samples, employing the WinBUGS program in MLwiN [49]. Any substantive differences among these methods are noted in the text.

## Results

### Hunger and HIV

**Fig 2:**
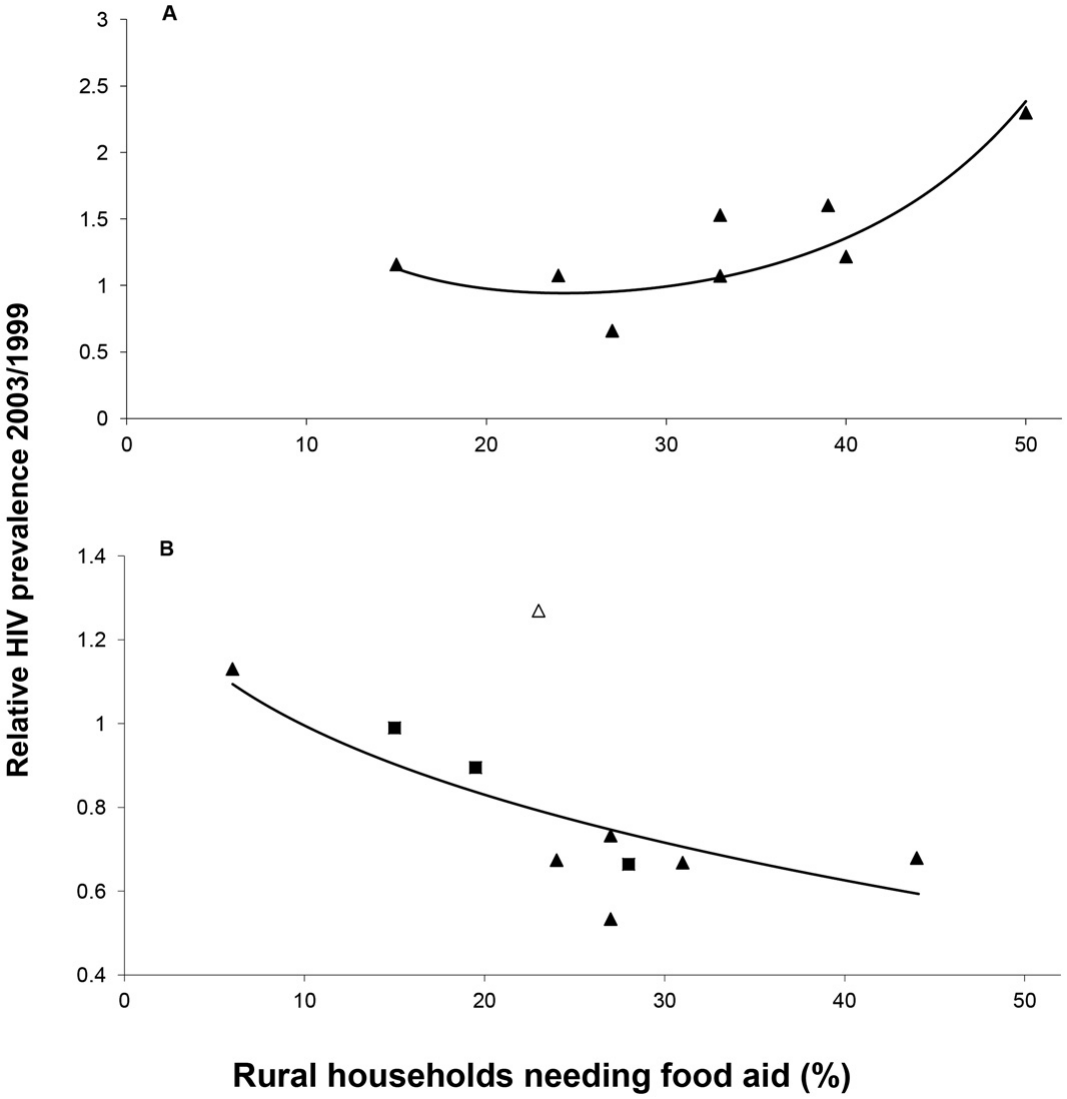
Change in HIV prevalence at antenatal sites through the famine in relation to rural hunger. The independent variable is the proportion of rural households estimated from a humanitarian survey to be in need of food assistance in December 2002—March 2003 in the district surrounding antenatal sites. The observed values of the dependent variable are the ratio of the HIV prevalence in women attending antenatal clinics in 2003, after the worst of the famine, to the prevalence in 1999/2000, prior to the rapid rise in food prices. The fitted values are from multilevel log binomial regression (model 1, Table 2). (A) Rural sites. The quadratic equation has a vertex at approximately 25%, implying little change in HIV around this level of hunger. (B) Non-rural sites (squares: cities, triangles: towns, open triangle: Nsanje, which is included in the analysis).

HIV prevalence increased 20.2% over the course of the famine at rural surveillance sites but declined 21.7% and 14.8% at semi-urban and urban sites, respectively (Table 1). The increase at rural sites was non-linearly related to the extent of rural hunger in the district, implying no increase in prevalence below a threshold of about 25% and a steep increase thereafter (model 1, Table 2; Fig 2). At non-rural sites, the decline in prevalence was negatively related to rural hunger with no evident non-linearity; urban and semi-urban sites followed a similar pattern. This regression includes one extreme outlier, the semi-urban site Nsanje (departure from predicted p < .001; see further below). At the rural and non-rural sites where hunger was greatest, model 1 estimates HIV prevalence increased 139% and declined 41%, respectively.

**Table 2:** Change in HIV prevalence at antenatal sites through the famine (from multilevel log-binomial regression).

			Model 1		Model 2	
	Variable	Category	Rural	Non-rural	Rural	Non-rural
**Site level**	Rural hunger	Linear	-0.231 (-0.470, 0.010) ^+^	-0.022 (-0.038, -0.006)**	-0.273 (-0.548, 0.002)^+^	-0.013 (-0.048, 0.013)
		Quadratic	0.004 (0.000, 0.008)* ^†^		0.004 (0.000, 0.008)*	
	Rural (dummy)		0.435 (0.161, 0.708) **			0.609 (0.060, 1.159)*
**Individual level**	Occupation	Farmer			-0.198 (-0.554, 0.159)	-0.304 (-0.433, -0.174)**
		Non-farmer (ref.)			0	0
	Age	< 25 yrs			-0.334 (-0.608, -0.061)*	-0.191 (-0.295, -0.086)**
		25 + yrs (ref.)			0	0
	Education	None (ref.)			0	0
		Primary			0.006 (-0.360, 0.348)	-0.031 (-0.168, 0.106)
		Secondary +			-0.126 (-0.796, 0.544)	0.161 (-0.007, 0.329^+^
**Interaction**	Occupation x rural hunger	Farmer			0.001 (-0.055, 0.057)	-0.024 (-0.043, -0.005) **
		Non-farmer (ref.)			0	0
	Age x rural hunger	< 25 yrs			-0.010 (-0.050, 0.030)	0.004 (-0.010, 0.018)
		25 + yrs (ref.)			0	0
	Education x rural hunger	None (ref.)			0	0
		Primary			0.031 (-0.020, 0.082)	-0.002 (-0.022, 0.019)
		Secondary +			0.042 (-0.053, 0.136)	0.002 (-0.022, 0.026)
**Intercept**			-0.249 (-0.369, -0.261) **		-0.041 (-0.217, 0.134)	
**Between site variance (s.e.)**			0.0299 (0.136)		0.040 (0.017)	
**Log likelihood**			-3802.6		-3725.0	

The dependent variable is the log of the relative risk of a woman being seropositive in 2003 versus 1999/2000. Data are coefficients (95% C.I.)

^+^ P < .10

* P < .05

** P < .01

^†^ Joint probability linear and quadratic < .05.

At rural sites, adding individual-level factors and interactions had little effect on the site-level relationship with hunger (model 2). Women under 25 years were at significantly less risk of infection relative to the pre-famine mean than older women but this risk and that of the other individual-level factors did not vary with the extent of hunger. At non-rural sites, women under 25 years and those who farmed were at significantly reduced risk while those with at least some secondary education were at increased risk relative to the pre-famine mean. Here, the risk for women farmers declined significantly as rural hunger rose: the log relative risk fell 0.037 per unit increase in hunger compared to a non-significant 0.013 for non-farmers, controlling for other factors.

The rural site results suggest an increased per capita risk of infection at levels of hunger above the threshold that was not concentrated in any particular group. This is consistent with the reports of women’s increased exposure to HIV through transactional sex, but not with more frequent early marriage whose effects would have been concentrated in the under 25 years age class. Factors that increased the likelihood of infection once exposed might also have contributed.

The non-rural site results suggest selective out-migration by farming women from rural areas. This is consistent with the evidence that the erosion of livelihoods and the effect of the price spike were most keenly felt by those dependent on farming; also that migration in search of food or work was common. Women who moved to towns and cities and who remained long enough to become pregnant (if they weren’t already when they moved) and attend antenatal clinics there would have reduced the HIV prevalence because they came from a lower risk environment: in 1999/2000, the probability of a farming women being HIV-positive at the rural sites was 10.6% (95% CI 6.2%–17.5%) but 22.0% (19.5%–24.8%) at the non-rural sites. This dilution would have increased the greater the level of hunger-induced migration. As discussed below, this effect is likely to have diminished with time.

Migration may explain the discrepant result at Nsanje. In 2005, a one-off surveillance site was established at a rural health centre in the district. Prevalence was 40.5% (n = 74, 95% CI 29.3%–51.7%), higher than at the district hospital in 1999/2000 (26.0%, 95% CI 21.8%–30.2%, Fisher exact P = 0.011), indeed the highest level ever recorded at an antenatal surveillance site in Malawi. If similar infection rates prevailed in rural Nsanje during the famine, migration from the villages would, as observed, have raised rather than lowered prevalence in the town.

### Hunger and migration

**Fig 3:**
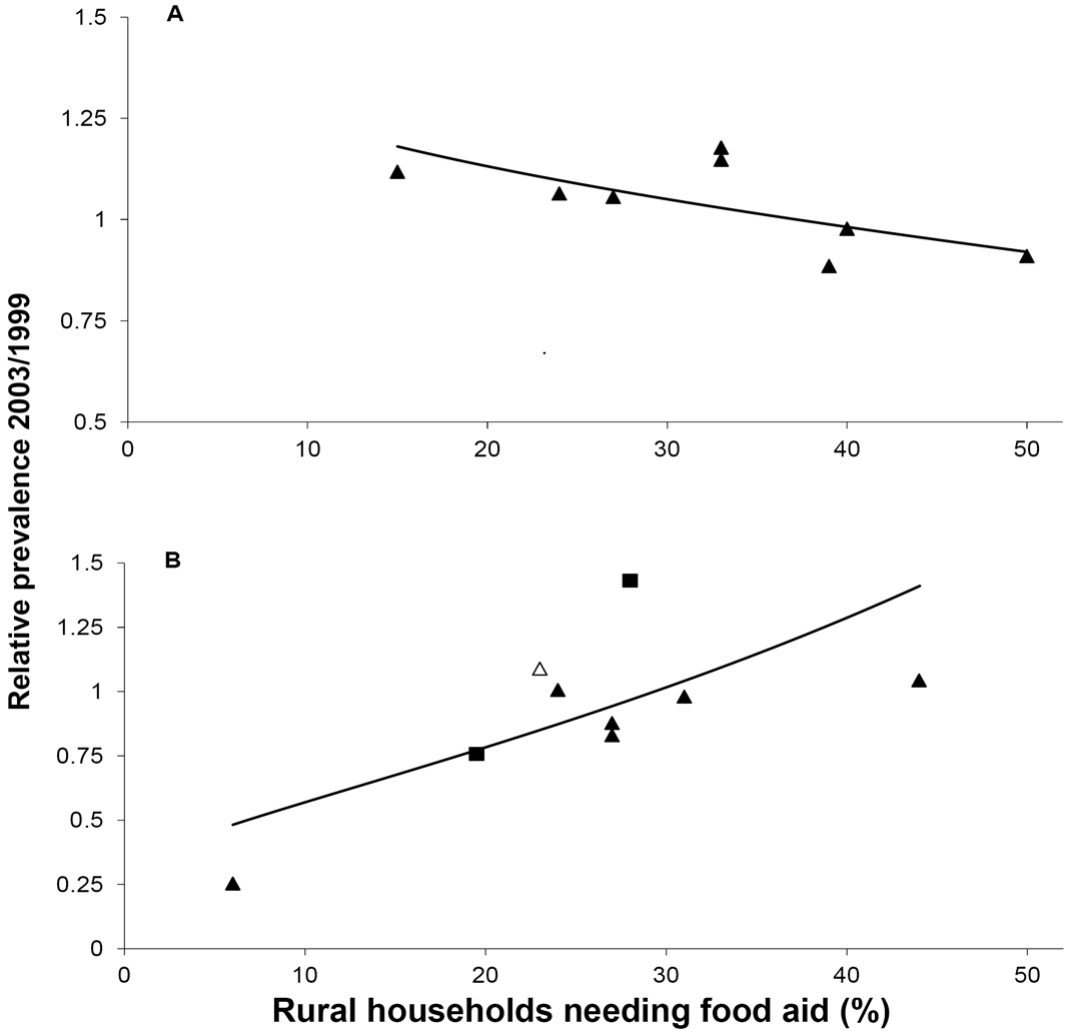
Change in prevalence of farmers at antenatal sites through the famine in relation to rural hunger. The independent variable is the proportion of rural households estimated from a humanitarian survey to be in need of food assistance in December 2002-March 2003 in the district surrounding antenatal sites. The observed values of the dependent variable are the ratio of the proportion of farmers among the women attending antenatal clinics in 2003, after the worst of the famine, to the proportion in 1999/2000, prior to the rapid rise in food prices. The fitted values are from multilevel log binomial regression (model 3, S2 Table). (A) Rural sites; (B) Non-rural sites (squares: cities, triangles: towns, open triangle: Nsanje, which is included in the analysis).

The suggestion that selective migration of farming women from rural areas underlies the decline in prevalence at non-rural sites is corroborated by the changing composition of the antenatal population over the course of the famine (S2 Table, Model 3, *see web appendix*; and Fig 3). As hunger rose in the surrounding district, the proportion of farmers declined at the rural antenatal sites but increased at the non-rural sites. Hunger pushed farmers into the towns and cities as it removed farmers from the villages.

Fig 3A and Model 3 indicate that at rural surveillance sites where hunger was relatively low, the proportion of farmers in 2003 was greater than in 1999/2000 (ratio>1). This is consistent with the accounts of villagers migrating to other rural as well as urban areas in search of food and work: districts where hunger was low would have been the most attractive.

Similarly, the fact that at the non-rural sites where rural hunger was low, the proportion of farmers in 2003 was less than in 1999/2000 (ratio<1, Fig 3B) could have resulted from women farmers resident in or near those towns leaving for the villages. However, this is unlikely a sufficient explanation because even at the highest levels of rural hunger the proportion of farmers in 2003 was not much greater than in 1999/2000. A simpler explanation, consistent with the hypotheses, is that, for many, leaving their villages was more than a temporary measure. A village woman attending an antenatal clinic in a town or city in 2003 who had decided to remain there would no longer give her occupation as “farmer”. In aggregate, such decisions would tend to depress the regression line. They would also lead to the overall decline in the proportion of farmers at non-rural, especially semi-urban sites evident in Table 1. Other evidence bearing on this is presented below.

Model 4 (S2 Table; *see web appendix*) indicates that at both rural and particularly non-rural sites, relative to the pre-famine mean, farmers were less educated than non-farmers but were not significantly older or younger. Neither of these relationships was found to vary with the level of rural hunger in the district.

However, markedly different results were obtained when Models 3 and 4 were estimated with logistic rather than log-binomial regression (Table 3). The negative relationship between rural hunger and the proportion of women farmers at rural antenatal sites is strengthened. Farmers were also found to be significantly younger and less educated than non-farmers at these sites. The interaction coefficients indicate that the proportion of farmers under 25 years decreased significantly relative to older farmers as rural hunger in the district rose: the log odds ratio declined 0.193 per unit increase in rural hunger for the former and 0.127 for the latter, controlling for individual-level factors and other interactions. Note however, that both coefficients are significant. This suggests that farmers under 25 years had a greater propensity to migrate in response to hunger than older farmers who in turn had a greater propensity to migrate than non-farmers.

**Table 3:** Change in farmer prevalence at antenatal sites through the famine (from multilevel logistic regression).

			Model 3		Model 4	
	Variable	Category	Rural	Non-rural	Rural	Non-rural
**Site level**	Rural hunger		-0.120 (-0.179, -0.062) **	0.052 (0.010, 0.094)*	-0.127 (-0.214, -0.040)**	0.068 (0.019, 0.117)**
	Rural (dummy)		0.692 (0.126, 1.259)*		0.823 (0.050, 1.596)*	
**Individual level**	Age	< 25 yrs			0.492 (0.113, 0.872)*	-0.052 (-0.191, 0.087)
		25 + yrs (ref.)			0	0
	Education	None (ref.)			0	0
		Primary			-0.833 (-1.391, -0.275)**	-0.236 (-0.418, -0.055)*
		Secondary +			-1.670 (-2.497, -0.842) **	-1.315 (-1.580, -1.051)**
**Interaction**	Age x rural hunger	< 25 yrs			-0.066 (-0.117, -0.016) **	-0.001 (-0.021, 0.019)
		25+ yrs (ref.)			0	0
	Education x rural hunger	None (ref.)			0	0
		Primary			0.044 (-0.028, 0.116)	-0.016 (-0.046, 0.013)
		Secondary +			0.026 (-0.078, 0.130)	-0.045 (-0.083, -0.007) *
**Intercept**			-0.125 (-0.438, 0.187)		0.224 (-0.121, 0.569)	
**Between site variance (s.e.)**			0.230 (0.092)		0.230 (0.092)	
**Log likelihood**			-3570.5		-3463.6	

The dependent variable is the log of the odds ratio of a woman being a farmer in 2003 versus 1999/2000. The data are coefficients (95% C.I.)

^+^ P < .10

* P < .05

** P < .01

At the non-rural sites, the proportion of farmers with no formal education increased relative to farmers with a primary or secondary and higher education as rural hunger in the district rose: the log odds ratio increased 0.068 per unit increase in rural hunger for those with no education, 0.052 for those with primary education and 0.023 for those with at least some secondary education, again controlling for individual-level factors and other interactions. Only the first two are significant. This suggests that immigrants to towns and cities were, relative to the receiving population, disproportionately less educated farmers and especially those with no formal education. There is no contradiction with the previous finding of a greater propensity to migrate in young farmers: that comparison was to women attending the rural antenatal clinics which differed in composition from the clients of non-rural clinics.

That the logistic version of model 4 reveals differences that the log-binomial form does not is expected: as noted earlier, the former is the more sensitive to change in a dependent variable whose frequency is relatively high. No substantial difference was evident between the log-binomial (Table 2) and logistic forms of model 1 and 2 (S1 Table; *see web appendix*): there the dependent variable, HIV prevalence, is generally less than 30%. The logistic regression results were confirmed by MCMC simulation.

**Fig 4:**
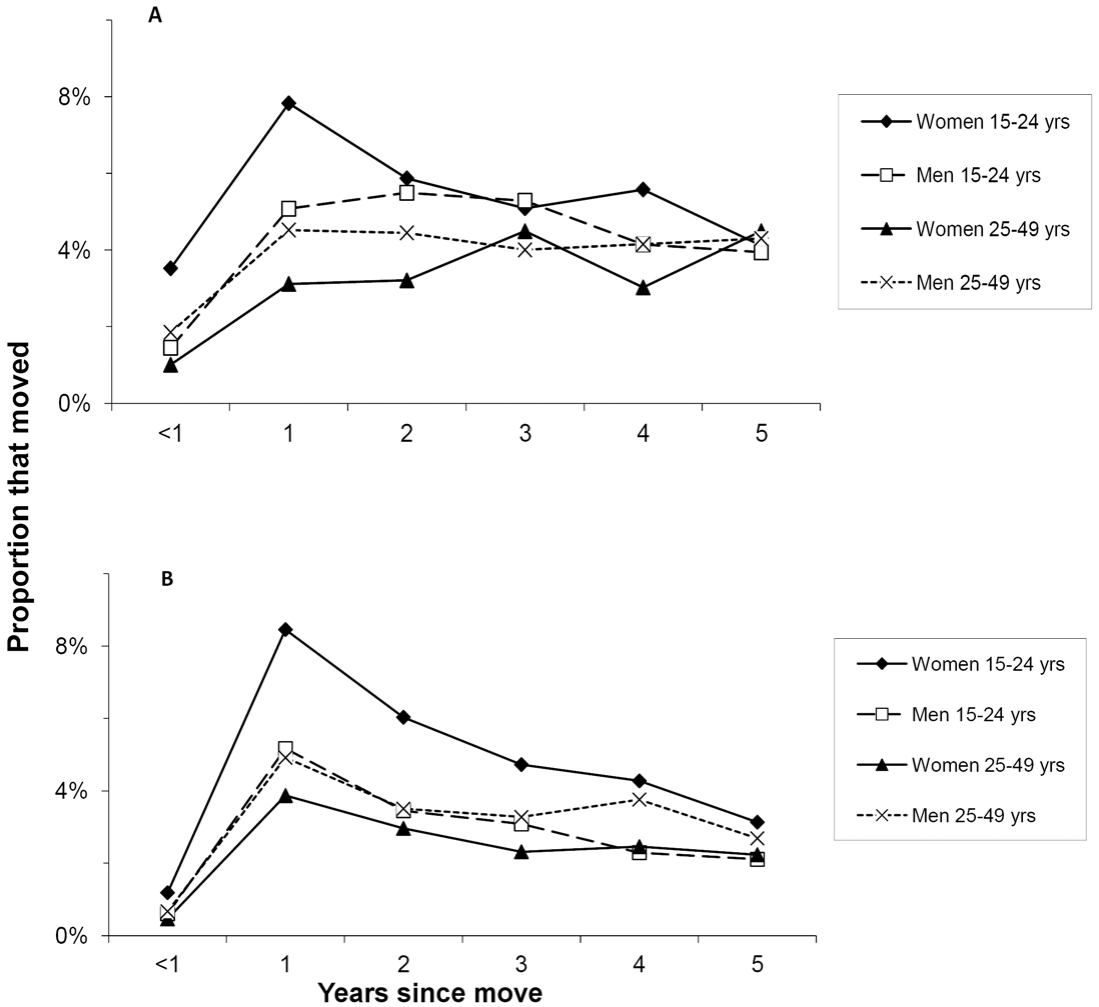
Prevalence of migration in Malawi, assessed in 2004 and early 2005. From the Integrated Household Survey [41]. (A) Rural to non-rural migration. Non-rural includes cities, towns and *bomas* (district administrative centres). N = 4,427. (B) Rural-to-rural migration. N = 17,722.

Data on migration from the 2004–05 Integrated Household Survey are presented in Fig 4. Across the country, 22.3% of women 15–24 years interviewed in towns and cities reported having moved to their place of residence from a rural area in the previous 3 years, roughly the period since the beginning of the famine. Among women 25–49 years, the corresponding proportion was 11.8% (χ^2^ = 41.3, P < .001); in men 15–24 years and 25–49 years it was 17.8% and 14.3%, respectively. Fig 4A also indicates that rural-to-non-rural migration in women 15–24 years peaked 1–2 years before the survey i.e. around the height of the food crisis in 2002–03. No such peak is evident in the other age-gender groups. The varying shape of these curves makes it less likely that some factor other than migration is responsible such as people recalling recent events better than more distant ones.

Fig 4B shows a similar pattern for rural-to-rural migration: greater in women 15–24 years than in women 25–49 years (20.4% vs. 9.6%, χ^2^ = 212.8, P < .001). In men 15–24 years and 25–49 years, the proportions were 12.3% and 12.4%, respectively. There is again a peak in migration 1–2 years before the survey that is most marked in women 15–24 years but also apparent in the other groups.

These results confirm the findings from the multilevel analysis of HIV prevalence and antenatal clinic composition: there is evidence of substantial migration from rural areas to both non-rural and other rural areas during the famine that involved women under 25 years of age significantly more than older women. Given that the IHS was conducted approximately a year after the 2003 antenatal surveillance round, the results also support the suggestion that for many women migration was more than a temporary response to hunger. There is no evidence for significant movement from towns and cities to the villages that might have affected these patterns (1.5% and 1.3% of 15–24 year old and 25–49 year old women, respectively).

### Testing assumptions

The validity of assessing the impact of the Malawi famine on HIV’s dynamics as a natural experiment may be undermined by two factors. First, it is possible that the levels of hunger calculated in late 2002–early 2003 reflected not a sharp, distinctive shock but pre-existing site-specific conditions that affected HIV’s dynamics in earlier periods as well. This is tested by regressing changes in prevalence at the same sites during the preceding 3 year period, 1996–1999/2000, against hunger in 2002–2003 in a single-level model. The coefficients for both the rural and non-rural sites were not significant (-0.032, 95% CI -0.078–0.015, n = 8, P = 0.17 and -0.033, 95% CI -0.087–0.020, n = 10, P = 0.20, respectively). Furthermore, the patterns of prevalence change across sites in 1996–1999/2000 and 1999/2000–2003 were not similar, as they would have been had the same factors, of whatever nature, driven change and been distributed in the same way. Paired by site, changes in prevalence in the two periods were not significantly correlated at rural, non-rural or all sites (r = -0.322, P = 0.44; r = -0.271, P = 0.45 and r = -0.298, P = 0.23, respectively). These findings are consistent with conditions during the famine being distinct from the preceding period, though as explained above, many of the famine’s social and economic causes had developed over a number of years.

The second factor that can undermine the experimental analysis is non-independence of the observational units, violating regression assumptions [50]. Migration between sites that preferentially involved people at greater or lesser risk of HIV is one notable way in which this could have been produced. Its importance is tested by calculating the spatial autocorrelation of the site residuals from model 1 [51]. No significant autocorrelation is found at any distance for all, rural and non-rural sites (S1 Fig, *see web appendix*).

This finding does not contradict the evidence that migration of young, rural women contributed to the decline in non-rural HIV prevalence because Model 1 calculates separate regression coefficients for rural and non-rural sites: origin and destination are statistically isolated. The non-significant spatial autocorrelation among rural sites is not surprising given the sparseness of the rural surveillance network: most sources of migrants to a given rural site would be from districts outside the network.

## Discussion

### Weighing the evidence

This analysis of the Malawi famine as a natural experiment suggests that it had a rapid and substantial effect on both HIV prevalence and demography across the country’s rural and non-rural areas. To frame and help readers weigh the evidence in support of this claim, I make use of the criteria that Hill [52] proposed should guide judgements of causation from evidence of association between environmental factors and disease. Fifty years on, they are still controversial and still widely cited.

#### Temporality

The timing of key events—the rise of food prices beginning in 2001, the reports of extreme coping measures including migration and transactional sex beginning in late 2001, the peak in migration in 2002–03 evident from IHS data and the substantial change in HIV prevalence found in the 2003 surveillance round—supports the suggested causal connection.

#### Strength of the association

The relative risks calculated at the rural and non-rural sites where hunger was greatest, 2.39 and 0.61, respectively, are substantial, considering that hunger was measured at the district rather than individual level.

#### Biological gradient

A dose-response in the relationship between hunger and change in HIV is evident at both rural and non-rural sites.

#### Plausibility

The mechanisms implicated in the hunger-HIV prevalence change relationship are biologically and socially plausible. The finding of an increased risk of HIV infection at the rural sites where hunger was greatest, broadly shared among age, occupation and education classes, is consistent with hunger leading women into increased involvement in transactional sex which raised their exposure to HIV and with depressed immune function increasing their risk of infection once exposed. As mentioned above, increased transactional sex was reported during the famine [22,23] and cross-sectional studies have found it more common among Zimbabwean, Batswana and Swazi women reporting food insecurity [5,6]. Its occurrence in other contexts is discussed below. Hunger may also have reduced women’s ability to insist non-cohabiting partners use a condom, as found in Brazil [53], however less than a quarter of rural women reported a condom was used in those situations before the famine [54].

The study implicates hunger-induced rural out-migration in the decline of HIV prevalence at non-rural sites. As noted above, such migration was a common response during the famine [16,22,33], as it was in earlier food crises in Malawi [55,56] and in famines elsewhere in Africa [37]. A simulation study suggests that increases in migration rates on the order of those observed during the famine (Fig 4) can drive substantial changes in HIV prevalence. Critical parameters are the difference in prevalence between migrating and receiving groups and their relative sizes [57]. In Malawi, the markedly lower rural prevalence and the much larger rural population (84% of the total versus 12% in the cities and 3% in the towns) [58] make it more plausible that rural out-migration could have driven the decline in non-rural prevalence.

Potential biases do not appear able to account for the study’s findings. For example, hunger might have reduced the likelihood of women attending antenatal clinics but to reproduce the observed patterns, HIV-negative women would had to be more affected than HIV-positive women in rural but not non-rural areas and farmers, especially poorly educated ones, would have had to be less affected than other women in non-rural but not rural areas. This does not seem plausible.

More plausible is that hunger could have hastened the death of HIV-positive people who are especially vulnerable to shortfalls in nutrition. They were at additional risk during the famine as households with chronically ill members, a proxy for AIDS, were found to be significantly more food insecure than other households [59]. This selective mortality would have depressed HIV prevalence particularly at the rural sites where hunger was greatest, diminishing to some extent the impact of hunger on HIV incidence.

#### Coherence

Agreement with independent evidence buttresses an imputed causal relationship. The conclusion that transactional sex apparently played a role in driving the increase in HIV prevalence at rural sites is consistent with contemporary accounts but no other corroborative evidence is available: it rests on a single multilevel regression. The case for the role of hunger-induced migration in the decline of prevalence at non-rural sites is considerably more robust because, in addition to the contemporary accounts, it is corroborated by four independent lines of evidence: the decline in prevalence is concentrated in farmers who were the most affected by hunger; the proportion of farmers in the antenatal population changes in a fashion consistent with out-migration at rural and in-migration at non-rural sites; and migration patterns calculated from the IHS data are consistent with those in the antenatal data in composition, timing and direction. Migration also offers a consistent explanation for the discrepant result, Nsanje, in Fig 2 where, unusually, rural prevalence was apparently greater than urban prevalence.

An alternative explanation for the decline in town and city prevalence post-2000 has been offered, based on changes in self-reported sexual behaviour (reduced number of partners and increased condom use among men): a model including these factors explained non-rural prevalence trends better than one that did not [60]. However, migration, which can confound `-based assessment of behaviour change using surveillance data [57], was not taken into account. The study could not explain the differences among non-rural sites in the rate of decline or the concomitant increase in rural prevalence.

The hunger hypothesis can account for this pattern of change and is amply corroborated. It may be a likelier explanation for the prevalence trends. A forthcoming paper assesses the two hypotheses in more detail. The policy implications are discussed below.

Hill [52] did not believe that the above or the other four criteria are each relevant in every case or required in order to support an inference of causation. **Analogies** from other, broadly similar relationships can make an imputed causal link more plausible but may not be available. **Specificity** of the relationship between exposure to a factor and risk of a disease can make the inference of causality more obvious and compelling. However, hunger, a structural determinant whose link with HIV risk is mediated through behaviour, is not an individually necessary cause. Below, I consider the contexts in which food crises might be expected to have epidemiological and demographic consequences comparable to those in the Malawi famine. Controlled **experiments** have important advantages but in the case of the hunger-HIV relationship are impossible to carry out for ethical and other reasons. Cross-sectional studies are constrained by the bi-directional linkages between hunger and HIV [6]. Natural experiments are, arguably, the best, possibly the only method available to explore that relationship, in its entirety, with sufficient realism. They have yielded invaluable insights in epidemiology [61,62] but remain an undervalued resource; many opportunities are not being exploited [63].

The remaining criterion is **consistency**: Has the relationship “been repeatedly observed by different persons, in different places, circumstances and times?” It has not, with the rigour epidemiology demands, for the reasons just alluded to.

The study has several limitations. The patterns it is able to discern are restricted by the density of available data. Trends in rural HIV prevalence rely on only 8 sites, the same number as for the towns whose population is far smaller. Hunger, the defining trait of the famine, is estimated from one survey in the final months of the famine whereas the experience over a longer period likely influenced coping responses. Similarly, the study’s ability to resolve migration patterns is limited by its reliance on hunger in the surrounding district as the independent variable. Migrants, particularly to the cities, may well have been attracted from much further. A more sophisticated spatial analysis might bring that out although neighbouring districts often shared socio-economic and agronomic characteristics and had similar levels of hunger. The methods employed also give no indication of the frequency of transient migration: women who returned to their villages before the 2003 HIV surveillance round or the 2004–05 IHS.

On the balance of the evidence, the claim that the Malawi famine appears to have had a rapid and substantial effect on both HIV prevalence and demography is reasonable. One can have greater confidence in hunger’s role in driving declining prevalence in the towns and cities than in its role in the increase in prevalence in the villages.

### Implications

If this interpretation of the evidence is correct, the famine’s consequences must be considered not just in terms of hunger, malnutrition and cholera but also in additional HIV infections and the illness and death they gave rise to; the lifelong costs of antiretroviral treatment; as well as the dislocated lives left by widespread migration that was far from voluntary and evidently not always temporary.

Migration clearly differed in one important respect from the patterns observed in response to a famine in 1949 [55] and to continuing food insecurity in the 1960–70s [56]. Then it was predominantly men who left in search of food or work whereas in 2001–03 the IHS data show young women were the largest group involved. This had important implications for women’s exposure to sexually transmitted infections. The situations of risk they now confronted were, for the most part, associated with their own movement rather than that of their male partners.

The magnitude of these risks can be estimated. A woman attending a rural antenatal clinic in 2003 who was a farmer, under 25 years of age and with less than a primary education had a 9.9% probability of being HIV positive (model 5). She had a probability of 12.9% if she attended a non-rural clinic and a 19.2% probability if her occupation changed to non-farmer, which appears to have been common. Migration nearly doubled her risk of infection, this suggests. This is in line with the widespread evidence, cited above, concerning the hazards a migrant confronts. But she may well have been at increased risk compared to a non-migrant, non-rural woman with similar characteristics because social isolation, added to poor education, would have left her with few skills or resources to avoid situations of infection risk in her new environment. Whatever the extent of this additional risk, it appears that the famine may have given rise to an increased burden of HIV infection not just among women in the villages but also among those forced into migration to towns and cities. The dilution of non-rural HIV prevalence by rural women migrating in distress may have been an effect that diminished with time, this suggests.

It is important to consider the relevance of this study’s findings to situations beyond Malawi in 2001–03. Available records from neighbouring countries in this period are less detailed but analysis of aggregate data from Zambia suggests a similar pattern of change in rural and non-rural HIV prevalence. Certainly, the situations of risk that have been implicated were widely reported in the region and persisted in Malawi in subsequent hungry seasons [25]. They are familiar in other developing regions dependent on rainfed agriculture such as semi-arid areas of India where seasonal, distress-linked migration is common [64]. Food crises that are not fundamentally seasonal in origin may provoke similar effects. Studies in South Asia and several parts of eastern and southern Africa indicate that the surge in global cereal prices in 2008 forced some people further into transactional sex and distress migration [65,66].

Whether a food access crisis produces epidemiologic and demographic consequences comparable to those that the Malawi famine appears to have provoked will depend on the frequency with which people are drawn into such situations of risk. At least two aspects of the Malawi context appear to have been critical in driving that frequency. First, the shocks that triggered the famine acted on a rural population whose livelihoods had become increasingly precarious; many had few options left other than the most extreme. Second, hunger was very unequally experienced. That inequality was central to the operation of several of the prevalent situations of infection risk. For example, economic and social disparities within rural communities drove transactional sex (a desperate seller, an able buyer) while geographic disparities among rural areas and between them and non-rural areas drove distress migration.

Poverty, hunger and inequalities are generally considered by public health professionals to be structural determinants of HIV infection and, as such, slow to change and essentially beyond the reach of near-term intervention in support of prevention [67]. This study’s findings suggest that these factors can in fact change rapidly and substantially, influencing HIV dynamics with little lag. When these factors are ignored, epidemic patterns may be misinterpreted, with critical implications for policy.

UNAIDS has cited the behaviour change study discussed above [60] as part of the evidence for widespread decline in HIV incidence in sub-Saharan Africa and other regions [68]. The evidence presented here suggests that rural outmigration, accelerated by hunger, may provide a credible alternative explanation for the non-rural decline in the first years of this century in Malawi. An important question then is whether rural out-migration has contributed materially to HIV declines elsewhere, which have often been concentrated in urban areas [69]. Conditions that make that plausible are frequently met in sub-Saharan Africa: prevalence is generally lower in rural areas (the mean ratio of rural to urban adult prevalence in 28 countries, taken from the most recent Demographic and Health Surveys [70], is 0.610; 95% CI 0.565–0.656), population is generally greater in rural than in urban areas and rural out-migration rates are volatile, responding to changing economic conditions and livelihood opportunities [71]. It is not evident that studies attributing declining HIV prevalence to sexual behaviour change have always followed Walker et al.’s advice to pay attention to migration if it is non-random with respect to HIV status or risk behaviour [57].

From a non-exhaustive literature search, eleven articles were identified that discuss the causes of HIV prevalence declines in the first decade of this century in southern Africa. All of these conclude that sexual behaviour change is the most plausible explanation, either alone or together with the increased AIDS-linked mortality that is expected as HIV epidemics mature [57]. Five studies make no mention of “migration” [60,69,72–74] and five of the six that do, do not consider the possibility that immigrants from lower prevalence areas or groups might have played a part in the decline [75–79]. One study in eastern Zimbabwe found that recent immigrants to the study areas had significantly lower prevalence than residents and that this contributed to the observed decline in prevalence [80]. However, the finding is not further discussed or mentioned in the paper’s title or abstract.

Returning to Malawi, the implications for policy are different if declining prevalence in towns and cities was predominantly the result of hunger-induced migration rather than behaviour change. Actions that bolster, rather than undermine, food and livelihood security would enable people to avoid situations of risk and escape HIV infection: these would complement effective behaviour change communication. Indeed, successful such efforts may already be doing that, yielding an as yet uncounted “prevention dividend”. The data themselves suggest one.

Over all districts, the prevalence of hunger in December 2002–March 2003, the independent variable in Figs 2 and 3, was significantly and negatively related to the prevalence of cassava cultivation [81]. In recent years, farmers in Malawi, Zambia and Mozambique, supported by research, have substantially expanded their production of cassava, a perennial crop more drought-tolerant than maize [82,83]. Cassava may also benefit households, whether or not they grow it, by damping demand for other foods and reducing pressure on prices. There was evidence of this in Malawi: the peak maize price in local markets during the famine was significantly and negatively related to the prevalence of cassava cultivation in the district [81].

The burden of other conditions, notably malnutrition, would have been eased by greater food security but this has yet to be assessed. Cassava’s contribution in subsequent food crises in 2005–06 and 2015 has also not been examined. But cassava is only one of many means to increase the diversity of agricultural, nutritional and livelihood options available to rural people, which underpins their resilience to climatic and market volatility. In an era of complex and, for many people, continuing crises, ensuring access to adequate and nutritious food will be critical to avert the worst health consequences.

## Supporting Information

S1 Fig Spatial autocorrelation of residuals from the multilevel regression in Model 1. No significant autocorrelation is evident at any distance.
(DOC)

S1 Table Change in HIV prevalence at antenatal sites through the famine (from multilevel logistic regression). The dependent variable is the log of the odds ratio of a woman being seropositive in 2003 versus 1999/2000.
(DOC)

S2 Table Change in farmer prevalence at antenatal sites through the famine (from multilevel log-binomial regression). The dependent variable is the log of the relative risk of a woman being a farmer in 2003 versus 1999/2000.
(DOC)

S1 Text Access to HIV surveillance data (DOC)

## References

[1] FAO, IFAD, WFP. The State of Food Insecurity in the World 2014 Strengthening the enabling environment for food security and nutrition. Rome: FAO 2014 Available from: http://www.fao.org/3/a-i4030e.pdf.

[2] Porter JR, Xie L, Challinor A, Cochrane K, Howden S, Iqbal M, et al Food Security and Food Production Systems. In: Climate Change 2014: Impacts, Adaptation, and Vulnerability. Contribution of Working Group II to the Fifth Assessment Report of the Intergovernmental Panel on Climate Change. Cambridge, UK, and New York: Cambridge University Press 2014.

[3] Vermeulen SJ, Aggarwal P, Ainslie A, Angelone C, Campbell BM, Challinor A, et al Options for support to agriculture and food security under climate change. Environmental Science & Policy. 2012; 15: 136–44.

[4] Gillespie S, Kadiyala S, Greener R. Is poverty or wealth driving HIV transmission? AIDS. 2007; 21: S5. 10.1097/01.aids.0000300531.74730.72 18040165

[5] Weiser SD, Leiter K, Bangsberg DR, Butler LM, Percy-de Korte F, Hlanze Z, et al Food insufficiency is associated with high-risk sexual behavior among women in Botswana and Swaziland. PLoS Medicine. 2007; 4: e260. 10.1371/journal.pmed.0040260 PMC2039764 17958460

[6] Pascoe S, Langhaug LF, Mavhu W, Hargreaves J, Jaffar S, Hayes R, et al Poverty, food insufficiency and HIV infection and sexual behaviour among young rural Zimbabwean women. PLoS ONE. 2015; 10: e0115290 10.1371/journal.pone.0115290 25625868 PMC4307980

[7] Susser M. Prenatal nutrition, birthweight, and psychological development: an overview of experiments, quasi-experiments, and natural experiments in the past decade. The American Journal of Clinical Nutrition. 1981; 34: 784–803. 7223694 10.1093/ajcn/34.4.784

[8] Sen A. Poverty and Famines: An Essay on Entitlement anti Deprivation. Oxford: Clarendon Press; 1981.

[9] Frankenberger T, Luther K, Fox K, Mazzeo J. Livelihood erosion through time: Macro and micro factors that influenced livelihood trends in Malawi over the last 30 years. Johannesburg: CARE Southern and Western Africa Regional Management Unit 2003 Available from: http://reliefweb.int/report/malawi/livelihood-erosion-through-time-macro-and-micro-factors-influenced-livelihood-trends

[10] Bryceson DF. The scramble in Africa: reorienting rural livelihoods. World Development. 2002; 30: 725–39.

[11] Mandala EC. The End of Chidyerano: a History of Food and Everyday Life in Malawi, 1860–2004: Praeger Publishers; 2005.

[12] Devereux S. The Malawi famine of 2002. IDS Bulletin. 2002; 33: 70–8.

[13] Shah MK, Osbourne N, Mbilizi T, Vilili G. Impact of HIV/AIDS on agricultural productivity and rural livelihoods in the Central Region of Malawi Lilongwe: CARE International 2002 Available from: http://books.google.co.uk/books/about/Impact_of_HIV_AIDS_on_Agricultural_Produ.html?id=jhgKAQAAMAAJ.

[14] Devereux S, Tiba Z. Malawi’s first famine, 2001–2002 In: Devereux S, editor. The New Famines: Why Famines Persist in an Era of Globalization. London: Routledge; 2007 p. 143–77.

[15] Ellis F, Kutengule M, Nyasulu A. Livelihoods and rural poverty reduction in Malawi. World Development. 2003; 31: 1495–510.

[16] World Food Programme. Full Report of the Real-Time Evaluation of WFP's Response to the Southern Africa Crisis, 2002–2003 (EMOP 10200). Rome: World Food Programme 2003. Available from: http://documents.wfp.org/stellent/groups/public/documents/reports/wfp022512.pdf.

[17] WHO. Cholera in Malawi: disease outbreak reported Geneva: WHO 2002. Available from: www.who.int/csr/don/2002_03_26/en/index.html

[18] MVAC. Malawi emergency food security assessment report (September). Lilongwe: Malawi Vulnerability Assessment Committee 2002. Available from: http://sarpn.org/documents/d0000046/index.php.

[19] MVAC. Malawi emergency food security assessment report (August). Lilongwe: Malawi Vulnerability Assessment Committee 2003. Available from: http://www.humanitarianresponse.info/system/files/documents/files/MAL_Food_Sec_Assessment_Report_Aug_2003.pdf.

[20] World Bank. Malawi Poverty and Vulnerability Assessment: Investing in Our Future. Washington 2007. Available from: https://openknowledge.worldbank.org/handle/10986/7909.

[21] Ngwira N, Bota S, Loevinsohn M. HIV/AIDS, agriculture and food security in Malawi. Lilongwe and The Hague: RENEWAL 2001. Available from: http://www.eldis.org/vfile/upload/1/document/1105/HIVAIDS.pdf.

[22] Kamowa O. Living in the abyss: hunger in Mchinji. Lilongwe: Save the Children (UK) 2002. Available from: http://www.eldis.org/go/home&id=23492&type=Document#.VN3rCC58oUo.

[23] Conroy AC, Blackie M, Whiteside A, Malewezi JC, Sachs JD. Poverty, AIDS, and hunger: Breaking the poverty trap in Malawi. London: Palgrave Macmillan; 2006.

[24] Bryceson DF, Fonseca J, Kadzandira J. Social pathways from the HIV/AIDS deadlock of disease, denial and desperation in rural Malawi. Lilongwe: CARE International 2004 Available from: http://www.sarpn.org/documents/d0000906/P1019-Malawi_Social-pathways_May2004.pdf.

[25] Bryceson DF. *Ganyu* casual labour, famine and HIV/AIDS in rural Malawi: casuality and casualty. Journal of Modern African Studies. 2006; 44: 173–202.

[26] Munthali A. The impact of the 2001/2002 hunger crisis on child labour and education: a case study of Kasungu and Mchinji Districts in Central Malawi In: Takane T, editor. Current Issues of Rural Development in Malawi. Chiba, Japan: Institute of Developing Economies; 2006 p. 23–65.

[27] Clark S. Early marriage and HIV risks in Sub-Saharan Africa. Studies in Family Planning. 2004; 35: 149–60. 15511059 10.1111/j.1728-4465.2004.00019.x

[28] Kishor S, Johnson K. Profiling domestic violence: a multi-country study. Calverton, Maryland: MEASURE DHS+ 2004. Available from: http://www.dhsprogram.com/publications/publication-OD31-Other-Documents.cfm.

[29] Garnett GP, Anderson RM. Sexually transmitted diseases and sexual behavior: insights from mathematical models. Journal of Infectious Diseases. 1996; 174: S150 8843245 10.1093/infdis/174.supplement_2.s150

[30] Morris M, Kretzschmar M. Concurrent partnerships and the spread of HIV. AIDS. 1997; 11: 641–8. 9108946 10.1097/00002030-199705000-00012

[31] Halperin DT, Epstein H. Concurrent sexual partnerships help to explain Africa's high HIV prevalence: implications for prevention. Lancet. 2004; 364: 4 15234834 10.1016/S0140-6736(04)16606-3

[32] Stillwaggon E. AIDS and the Ecology of Poverty. New York: Oxford University Press; 2006.

[33] Devereux S, Chilowa W, Kadzandira J, Mvula P, Tsoka M. Malawi food crisis impact survey: a research report on the impacts, coping behaviours and formal responses to the food crisis in Malawi of 2001/02. Brighton, UK and Lilongwe, Malawi: Institute of Development Studies and Centre for Social Research 2003 Available from: http://citeseerx.ist.psu.edu/viewdoc/summary?doi=10.1.1.359.6681.

[34] Pison G, Le Guenno B, Lagarde E, Enel C, Seck C. Seasonal migration: a risk factor for HIV infection in rural Senegal. Journal of Acquired Immune Deficiency Syndromes. 1993; 6: 196 8433284

[35] Decosas J, Kane F, Anarfi JK, Sodji K, Wagner H. Migration and AIDS. Lancet. 1995; 346: 826 7674750 10.1016/s0140-6736(95)91631-8

[36] Lurie MN, Williams BG, Zuma K, Mkaya-Mwamburi D, Garnett GP, Sturm AW, et al The impact of migration on HIV-1 transmission in South Africa: a study of migrant and nonmigrant men and their partners. Sexually Transmitted Diseases. 2003; 30: 149 12567174 10.1097/00007435-200302000-00011

[37] Von Braun J, Teklu T, Webb P. Famine in Africa: Causes, Responses, and Prevention: Johns Hopkins University Press Baltimore, Maryland; 1999.

[38] Susser M. What is a cause and how do we know one? A grammar for pragmatic epidemiology. American Journal of Epidemiology. 1991 4 1, 1991; 133: 635–48. 2018019 10.1093/oxfordjournals.aje.a115939

[39] Itano N. Man-made food crisis grips southern Africa: Malawi, Zambia, and Zimbabwe are on the verge of a famine exacerbated by poor management and corruption. Christian Science Monitor. 2012 5 15, 2002.

[40] RENEWAL. HIV/AIDS and food crises in southern Africa: A call for proposals. The Hague and Washington: Regional Network on HIV/AIDS, Rural Livelihoods and Food Security 2003. Available from: http://sarpn.org/documents/d0000401/RENEWAL_Proposals.pdf.

[41] National Statistical Office. Integrated Household Survey 2004–2005. Zomba, Malawi: National Statistical Office 2005. Available from: http://go.worldbank.org/SNV7M0LTI0.

[42] National AIDS Commission. HIV sentinel surveillance report 2003. Lilongwe: Ministry of Health and Population 2003. Available from: http://gametlibrary.worldbank.org/FILES/678_HIV%20Seroprevalence%20survey%20Malawi%202003.pdf.

[43] Gouws E, Mishra V, Fowler TB. Comparison of adult HIV prevalence from national population-based surveys and antenatal clinic surveillance in countries with generalised epidemics: implications for calibrating surveillance data. Sexually Transmitted Infections. 2008 8 1, 2008; 84: i17–i23. 10.1136/sti.2008.030452 18647861 PMC2569190

[44] Montana LS, Mishra V, Hong R. Comparison of HIV prevalence estimates from antenatal care surveillance and population-based surveys in sub-Saharan Africa. Sexually Transmitted Infections. 2008 8 1, 2008; 84: i78–i84. 10.1136/sti.2008.030106 18647871 PMC2569136

[45] Ukoumunne OC, Thompson SG. Analysis of cluster randomized trials with repeated cross-sectional binary measurements. Statistics in Medicine. 2001; 20: 417–33. 11180311 10.1002/1097-0258(20010215)20:3<417::aid-sim802>3.0.co;2-g

[46] Rabe-Hesketh S, Skrondal A, Pickles A. Maximum likelihood estimation of limited and discrete dependent variable models with nested random effects. Journal of Econometrics. 2005; 128: 301–23.

[47] Lee J, Tan C, Chia K. A practical guide for multivariate analysis of dichotomous outcomes. Ann Acad Med Singapore. 2009; 38: 714–9. 19736577

[48] Greenland S. Interpretation and choice of effect measures in epidemiologic analyses. Am J Epidemiol. 1987; 125: 761–8. 3551588 10.1093/oxfordjournals.aje.a114593

[49] Browne W. MCMC estimation in MLwiN, Version 2.10 Bristol, UK: Centre for Multilevel Modelling, University of Bristol 2009. Available from: http://seis.bris.ac.uk/~frwjb/bill.html.

[50] Schabenberger O, Gotway CA. Statistical Methods for Spatial Data Analysis. Boca Raton: CRC Press; 2004.

[51] Rangel TF, Diniz-Filho JAF, Bini LM. SAM: a comprehensive application for spatial analysis in macroecology. Ecography. 2010; 33: 46–50.

[52] Hill AB. The environment and disease: association or causation? Proceedings of the Royal Society of Medicine. 1965; 58: 295–300. 14283879 10.1177/003591576505800503 PMC1898525

[53] Tsai AC, Hung KJ, Weiser SD. Is food insecurity associated with HIV risk? Cross-sectional evidence from sexually active women in Brazil. PLoS Medicine. 2012; 9: e1001203 10.1371/journal.pmed.1001203 22505852 PMC3323512

[54] National Statistical Office, ORC Macro. Malawi Demographic and Health Survey 2000. Zomba, Malawi and Calverton, Maryland, USA: National Statistical Office and ORC Macro 2001. Available from: http://www.dhsprogram.com/publications/publication-FR123-DHS-Final-Reports.cfm.

[55] Vaughan M. The story of an African famine: Gender and famine in twentieth-century Malawi. Cambridge: Cambridge Univ Press; 2007.

[56] Christiansen R. The pattern of internal migration in response to structural change in the economy of Malawi 1966–77. Development and Change. 1984; 15: 125–51.

[57] Walker PT, Hallett TB, White PJ, Garnett GP. Interpreting declines in HIV prevalence: impact of spatial aggregation and migration on expected declines in prevalence. Sexually Transmitted Infections. 2008; 84: ii42–ii8. 10.1136/sti.2008.029975 18799492

[58] National Statistical Office. Population and housing census: final report. Zomba, Malawi: National Statistical Office. 1998 Available from: http://www.nsomalawi.mw/component/content/article/8-reports/127-1998-population-and-housing-census.html.

[59] VAC. Towards identifying impacts of HIV/AIDS on food insecurity in southern Africa and implications for response. Harare: Vulnerability Asessment Committee, Food Agricukture and Natural Resources, Southern African Development Community 2003 Available from: http://sarpn.org/documents/d0000321/P315_SADC_FANR_Report.pdf.

[60] Bello G, Simwaka B, Ndhlovu T, Salaniponi F, Hallett TB. Evidence for changes in behaviour leading to reductions in HIV prevalence in urban Malawi. Sexually Transmitted Infections. 2011; 87: 296–300. 10.1136/sti.2010.043786 21429896 PMC3252594

[61] Stein Z, Susser M, Saenger G, Marolla F. Famine and human development: The Dutch hunger winter of 1944–1945. New York: Oxford University Press; 1975.

[62] Lumey L, Stein AD, Susser E. Prenatal famine and adult health. Annual Review of Public Health. 2011; 32: 237–62. 10.1146/annurev-publhealth-031210-101230 21219171 PMC3857581

[63] Loevinsohn M. Natural experiments: An under-appreciated evaluation resource? Brighton, UK: Centre for Development Impact, Institute of Development Studies 2013 Available from: http://opendocs.ids.ac.uk/opendocs/bitstream/handle/123456789/3142/CDIPP02_Natural%20Experiments_AnUnderappreciatedEvaluationResource.pdf?sequence=1.

[64] George CK, Kavitha K, Reddy NS, Srikanthi B. Social context assessment for HIV/AIDS prevention programmes in Andhra Pradesh. Hyderabad, India: Institute of Health Systems 2005 Available from: http://www.ihs.org.in/Publications/reports.htm.

[65] Gillespie S, Jere P, Msuya J, Drimie S. Food prices and the HIV response: findings from rapid regional assessments in eastern and southern Africa in 2008. Food Security. 2009; 1: 261–9.

[66] Hossain N, McGregor JA. A ‘lost generation’? Impacts of complex compound crises on children and young people. Development Policy Review. 2011; 29: 565–84.

[67] Piot P, Bartos M, Larson H, Zewdie D, Mane P. Coming to terms with complexity: a call to action for HIV prevention. The Lancet. 2008; 372: 845–59. 10.1016/S0140-6736(08)60888-0 18687458

[68] UNAIDS. World AIDS Day Report 2011. Geneva: UNAIDS 2011. Available from: http://www.unaids.org/sites/default/files/en/media/unaids/contentassets/documents/unaidspublication/2011/JC2216_WorldAIDSday_report_2011_en.pdf.

[69] Hallett TB, Aberle-Grasse J, Bello G, Boulos L, Cayemittes M, Cheluget B, et al Declines in HIV prevalence can be associated with changing sexual behaviour in Uganda, urban Kenya, Zimbabwe, and urban Haiti. Sexually Transmitted Infections. 2006; 82: i1–i8. 16581753 10.1136/sti.2005.016014 PMC1693572

[70] Demographic and Health Surveys. AIDS Indicator Survey. [Internet] [20 February 2015]; Available from: http://dhsprogram.com/What-We-Do/Survey-Types/AIS.cfm.

[71] Potts D. Whatever happened to Africa’s rapid urbanisation? World Economics. 2012; 13: 17–29.

[72] Gouws E, Stanecki KA, Lyerla R, Ghys PD. The epidemiology of HIV infection among young people aged 15–24 years in southern Africa. Aids. 2008; 22: S5–S16. 10.1097/01.aids.0000341773.86500.9d 19033755

[73] Bello GA, Chipeta J, Aberle-Grasse J. Assessment of trends in biological and behavioural surveillance data: is there any evidence of declining HIV prevalence or incidence in Malawi? Sexually Transmitted Infections. 2006; 82: i9–i13. 16581763 10.1136/sti.2005.016030 PMC2593073

[74] Stringer EM, Chintu NT, Levy JW, Sinkala M, Chi BH, Muyanga J, et al Declining HIV prevalence among young pregnant women in Lusaka, Zambia. Bulletin of the World Health Organization. 2008; 86: 697–702. 18797645 10.2471/BLT.07.045260 PMC2649483

[75] Zulu LC, Kalipeni E, Johannes E. Analyzing spatial clustering and the spatiotemporal nature and trends of HIV/AIDS prevalence using GIS: the case of Malawi, 1994–2010. BMC Infectious Diseases. 2014; 14: 285 10.1186/1471-2334-14-285 24886573 PMC4057596

[76] Hargrove JW, Humphrey JH, Mahomva A, Williams BG, Chidawanyika H, Mutasa K, et al Declining HIV prevalence and incidence in perinatal women in Harare, Zimbabwe. Epidemics. 2011; 3: 88–94. 10.1016/j.epidem.2011.02.004 21624779

[77] Michelo C, Sandøy I, Dzekedzeke K, Siziya S, Fylkesnes K. Steep HIV prevalence declines among young people in selected Zambian communities: population-based observations (1995–2003). BMC Public Health. 2006; 6: 279 17096833 10.1186/1471-2458-6-279 PMC1660545

[78] Sandøy IF, Kvåle G, Michelo C, Fylkesnes K. Antenatal clinic-based HIV prevalence in Zambia: Declining trends but sharp local contrasts in young women. Tropical Medicine & International Health. 2006; 11: 917–28. 16772014 10.1111/j.1365-3156.2006.01629.x

[79] Gregson S, Gonese E, Hallett TB, Taruberekera N, Hargrove JW, Lopman B, et al HIV decline in Zimbabwe due to reductions in risky sex? Evidence from a comprehensive epidemiological review. International Journal of Epidemiology. 2010; 39: 1311–23. 10.1093/ije/dyq055 20406793 PMC2972436

[80] Gregson S, Garnett GP, Nyamukapa CA, Hallett TB, Lewis JJ, Mason PR, et al HIV decline associated with behavior change in eastern Zimbabwe. Science. 2006; 311: 664–6. 16456081 10.1126/science.1121054

[81] Loevinsohn M. Seasonal hunger, the 2001–03 famine and the dynamics of HIV in Malawi In: Devereux S., Sabates-Wheeler R., Longhurst R, editors. Seasonality, Rural Livelihoods and Development. London: Earthscan; 2011.

[82] Haggblade S, Longabaugh S, Tschirley D. Spatial patterns of food staple production and marketing in South East Africa: implications for trade policy and emergency response. East Lansing: Michigan State University 2009 Contract No.: 100. Available from: https://ideas.repec.org/p/ags/midiwp/54553.htm.

[83] Rusike J, Mahungu N, Jumbo S, Sandifolo V, Malindi G. Estimating impact of cassava research for development approach on productivity, uptake and food security in Malawi. Food Policy. 2010; 35: 98–111.

