# Supplementary material for: The 2001-03 Famine and the Dynamics of HIV in Malawi: A Natural Experiment
Source: PLoS One. 2015 Sep 2;10(9):e0135108. doi: 10.1371/journal.pone.0135108 (PMC4558031; doi:10.1371/journal.pone.0135108)
Supplement: S1 Fig — No significant autocorrelation is evident at any distance. (DOC) [file pone.0135108.s001.doc]

S1 Fig. Spatial autocorrelation of residuals from the multilevel regression in Model 1.

All sites (n=18)

Kilometerss

Rural sites (n=8)

Kilometerss

Non-rural sites (n=10)

Kilometerss

No significant autocorrelation is evident at any distance. Calculated using the SAM software. Rangel TF, Diniz-Filho JAF, Bini LM (2010) SAM: a comprehensive application for spatial analysis in macroecology. Ecography 33: 46-50.
