# Supplementary material for: The 2001-03 Famine and the Dynamics of HIV in Malawi: A Natural Experiment
Source: PLoS One. 2015 Sep 2;10(9):e0135108. doi: 10.1371/journal.pone.0135108 (PMC4558031; doi:10.1371/journal.pone.0135108)
Supplement: S1 Table — The dependent variable is the log of the odds ratio of a woman being seropositive in 2003 versus 1999/2000. (DOC) [file pone.0135108.s002.doc]

**S1 Table. Change in HIV prevalence at antenatal sites through the famine (from multilevel logistic regression).**

|  |  |  | **Model 1** | | **Model 2** | |
| --- | --- | --- | --- | --- | --- | --- |
|  | **Variable** | **Category** | **Rural** | **Non-rural** | **Rural** | **Non-rural** |
| **Site level** | Rural hunger | Linear | -0.247 (-0.470, 0.010) | -0.029 (-0.0497, -0.008)** | -0.309 (-0.606, -0.013)* | -0.016 (-0.043, 0.012) |
|  |  | Quadratic | 0.004 (-0.000, 0.008) ^+ †^ |  | 0.005 (0.001, 0.009)* ^§^ |  |
|  | Rural (dummy) |  | 0.531 (0.183, 0.839)** |  | 0.756 (0.188, 1.324)** |  |
| **Individual level** | Occupation | Farmer |  |  | -0.194 (-0.551, 0.163) | -0.307 (-0.436, -0.177)** |
|  |  | Non-farmer (ref.) |  |  | 0 | 0 |
|  | Age | < 25 yrs |  |  | -0.335 (-0.608, -0.061)* | -0.190 (-0.295, -0.086)** |
|  |  | 25 + yrs (ref.) |  |  | 0 | 0 |
|  | Education | None (ref.) |  |  | 0 | 0 |
|  |  | Primary |  |  | -0.007 (-0.361, 0.348) | -0.032 (-0.169, 0.105) |
|  |  | Secondary + |  |  | -0.126 (-0.796, 0.544) | 0.160 (-0.008, 0.329)^+^ |
| **Interaction** | Occupation x rural hunger | Farmer |  |  | 0.001 (-0.056, 0.057) | -0.024 (-0.043, -0.006)** |
|  |  | Non-farmer (ref.) |  |  | 0 | 0 |
|  | Age x rural hunger | < 25 yrs |  |  | -0.010 (-0.050, 0.030) | 0.004 (-0.010, 0.018) |
|  |  | 25 + yrs (ref.) |  |  | 0 | 0 |
|  | Education x rural hunger | None (ref.) |  |  | 0 | 0 |
|  |  | Primary |  |  | 0.031 (-0.020, 0.082) | -0.002 (-0.023, 0.019) |
|  |  | Secondary + |  |  | 0.042 (-0.053, 0.136) | 0.002 (-0.023, 0.026) |
| **Intercept** | | | -0.323 (-0.478, -0.168)** | | -0.359 (-0.548, -0.170)** | |
| **Between site variance (s.e.)** | | | 0.051 (.023) | | 0.053 (0.022) | |
| **Log likelihood** | | | -3803.4 | | -3727.1 | |

The dependent variable is the log of the odds ratio of a woman being seropositive in 2003 versus 1999/2000. Coefficients (95% C.I.)

^+^ P < .10,* P < .05, ** P < .01, ^†^ Joint probability linear and quadratic < .05,  ^§^ Joint probability linear and quadratic < .10
