# Supplementary material for: The 2001-03 Famine and the Dynamics of HIV in Malawi: A Natural Experiment
Source: PLoS One. 2015 Sep 2;10(9):e0135108. doi: 10.1371/journal.pone.0135108 (PMC4558031; doi:10.1371/journal.pone.0135108)
Supplement: S2 Table — The dependent variable is the log of the relative risk of a woman being a farmer in 2003 versus 1999/2000. (DOC) [file pone.0135108.s003.doc]

**S2 Table. Change in farmer prevalence at antenatal sites through the famine (from multilevel log-binomial regression).**

|  |  |  | **Model 3** | | **Model 4** | |
| --- | --- | --- | --- | --- | --- | --- |
|  | **Variable** | **Category** | **Rural** | **Non-rural** | **Rural** | **Non-rural** |
| **Site level** | Rural hunger |  | -0.011 (-0.024, 0.001)^+^ | 0.039 (0.020, 0.058)** | -0.005 (-0.019, 0.010) | 0.041 (0.019, 0.064)** |
|  | Rural (dummy) |  | 0.227 (0.059, 0.395)** |  | 0.035 (-0.166, 0.236) |  |
| **Individual level** | Age | < 25 yrs |  |  | 0.025 (-0.039, 0.088) | -0.034 (-0.117, 0.048) |
|  |  | 25+ yrs (ref.) |  |  | 0 | 0 |
|  | Education | None (ref.) |  |  | 0 | 0 |
|  |  | Primary |  |  | -0.030 (-0.078, 0.019) | -0.096 (-0.194, 0.002)^+^ |
|  |  | Secondary + |  |  | -0.205 (-0.373, -0.037)* | -0.824 (-1.015, -0.634)** |
| **Interaction** | Age x rural hunger | < 25 yrs |  |  | -0.004 (-0.010, 0.0002) | -0.000 (-0.010, 0.011) |
|  |  | 25+ yrs (ref.) |  |  | 0 | 0 |
|  | Education x rural hunger | None (ref.) |  |  | 0 | 0 |
|  |  | Primary |  |  | -0.001 (-0.006, 0.003) | -0.006 (-0.019, 0.007) |
|  |  | Secondary + |  |  | -0.015 (-0.039, 0.009) | -0.008 (-0.032, 0.016) |
| **Intercept** | | | -0.134 (-0.277, 0.008) ^+^ | | 0.046 (-0.114, 0.205) | |
| **Between site variance (s.e.)** | | | 0.040 (0.019) | | 0.046 (0.022) | |
| **Log likelihood** | | | -3575.6 | | -3479.6 | |

The dependent variable is the log of the relative risk of a woman being a farmer in 2003 versus 1999/2000.

Data are coefficients (95% C.I.)

^+^ P < .10,* P < .05, ** P < .01
