# Supplementary material for: The 2001-03 Famine and the Dynamics of HIV in Malawi: A Natural Experiment
Source: PLoS One. 2015 Sep 2;10(9):e0135108. doi: 10.1371/journal.pone.0135108 (PMC4558031; doi:10.1371/journal.pone.0135108)
Supplement: S1 Text — (DOC) [file pone.0135108.s004.doc]

S1 Text. **Access to HIV surveillance data**

The data from the 1999/2000 and 2003 HIV antenatal surveillance surveys can be requested by contacting:

Public Health Institute of Malawi

**Disease Surveillance, Prevention, Response and Control Division**
P/Bag 65
Lilongwe, MALAWI
+265 (0) 111 737 255
[**info@malawipublichealth.org**](http://malawipublichealth.org/contactus/info@phim.org)

[**http://malawipublichealth.org/epidemiology/**](http://malawipublichealth.org/epidemiology/)
